# MLP and CARP are linked to chronic PKCα signalling in dilated cardiomyopathy

**DOI:** 10.1038/ncomms12120

**Published:** 2016-06-29

**Authors:** Stephan Lange, Katja Gehmlich, Alexander S. Lun, Jordan Blondelle, Charlotte Hooper, Nancy D. Dalton, Erika A. Alvarez, Xiaoyu Zhang, Marie-Louise Bang, Yama A. Abassi, Cristobal G. dos Remedios, Kirk L. Peterson, Ju Chen, Elisabeth Ehler

**Affiliations:** 1 School of Medicine, University of California, San Diego, La Jolla CA-92093, USA; 2 BHF Centre of Research Excellence Oxford, Division of Cardiovascular Medicine, Radcliffe Department of Medicine, University of Oxford, Oxford OX3 9DU, UK; 3 ACEA Biosciences, 6779 Mesa Ridge Rd #100, San Diego, CA-92121, USA; 4 Institute of Genetic and Biomedical Research, UOS Milan, National Research Council, Rozzano (Milan) 20089, Italy; 5 Humanitas Clinical and Research Center, Rozzano (Milan) 20089, Italy; 6 Bosch Institute, Department of Anatomy, University of Sydney, Sydney 2006, Australia; 7 BHF Centre of Research Excellence at King's College London, Cardiovascular Division and Randall Division of Cell and Molecular Biophysics, London SE1 1UL, UK

## Abstract

MLP (muscle LIM protein)-deficient mice count among the first mouse models for dilated cardiomyopathy (DCM), yet the exact role of MLP in cardiac signalling processes is still enigmatic. Elevated PKCα signalling activity is known to be an important contributor to heart failure. Here we show that MLP directly inhibits the activity of PKCα. In end-stage DCM, PKCα is concentrated at the intercalated disc of cardiomyocytes, where it is sequestered by the adaptor protein CARP in a multiprotein complex together with PLCβ1. In mice deficient for both MLP and CARP the chronic PKCα signalling chain at the intercalated disc is broken and they remain healthy. Our results suggest that the main role of MLP in heart lies in the direct inhibition of PKCα and that chronic uninhibited PKCα activity at the intercalated disc in the absence of functional MLP leads to heart failure.

## Abstract (web summary)

Altered function of the muscle LIM protein (MLP) causes dilated cardiomyopathy in mice and humans. Lange *et al*. explain the molecular role of MLP in the heart by showing that it affects the signalling complex at the intercalated discs of failing hearts that consists of PKCα, PLCβ1 and CARP by inhibiting PKCα auto-phosphorylation and function.

## Graphical abstract

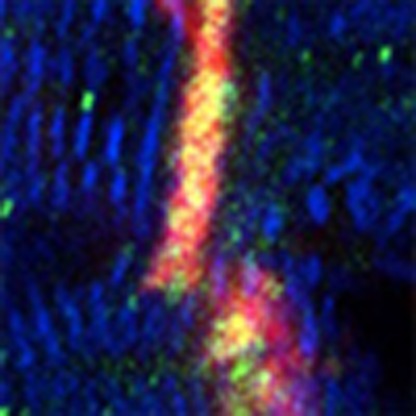

MLP (Muscle LIM protein, encoded by the *Csrp3* gene) was initially discovered as a protein up-regulated in skeletal muscle following denervation^1^. It was subsequently shown to be expressed only in the heart and in adult slow-twitch skeletal muscle, and suggested to play a role during muscle differentiation^1^,^2^. MLP consists of two LIM domains, structural domains composed of two zinc fingers, which are well known for their role in protein–protein interactions^3^,^4^. Among the many binding partners that were described for MLP are the cytoskeletal proteins actin, α-actinin, N-RAP, telethonin (T-cap) and spectrin as well as the skeletal muscle transcription factors MyoD, MRF4 and myogenin^5^,^6^,^7^,^8^,^9^. Based on these interactions and the presence of a nuclear localization signal it was proposed that MLP acts as a signalling protein between the myofilaments or the cytoplasm, and the nucleus in myocytes, which is responsive to pharmacological or mechanical stimuli^10^. Pathological mutations in MLP can lead to familial hypertrophic cardiomyopathy (HCM)^11^ or dilated cardiomyopathy (DCM)^8^.

Mice deficient for MLP (MLP knockouts) count among the first published models for DCM in a genetically manipulated animal^12^. They show all the anatomical and physiological hallmarks of DCM, and present with up-regulated expression levels of classical biomarkers for hypertrophy such as ANF (atrial natriuretic factor), BNP (brain natriuretic peptide) and β-myosin heavy chain as well as stress markers such as CARP (Cardiac-specific ankyrin repeat protein, CARP1/Ankrd1)^12^. While MLP knockout mice have been used by many laboratories as a mouse model to investigate DCM, the exact role that MLP plays in myocytes remains unclear. It was proposed that MLP could act as a mechanosensor at the Z-disc transmitting stress signals to the nucleus^8^,^10^,^13^. While signalling roles of MLP in the heart are well characterized, its function as a mechanosensor is less clear. Considering its molecular structure and subcellular localization, it is not obvious how a 20 kDa protein that exclusively consists of LIM domains can sense changes in mechanical force. Given that LIM domains have well-known protein–protein interaction interfaces^3^,^4^, it seems more likely that MLP functions in signal transmission rather than as a direct mechanosensor. Additionally, the exclusive Z-disc localization has been challenged, since several groups have reported a more widespread distribution throughout several subcellular compartments in myocytes both for endogenous and transfected MLP, including the nucleus, plasma membrane, cytoplasm, cytoskeleton and myofibrillar localizations other than the Z-disc^6^,^10^,^11^,^13^,^14^.

Over the years numerous rescue models were published using MLP knockout mice including double knockout mice with the SERCA-2A (Sarcoplasmic reticulum Ca^++^ ATPase) regulator phospholamban^15^ and overexpression of calcineurin^16^. Other MLP knockout rescue reports involve inhibition of adrenergic signalling^17^,^18^ and interference with PKCα (Protein Kinase C) signalling^19^,^20^.

Increased PKCα expression and activity are well established in end stage heart failure models in rodents (for recent reviews see^21^,^22^). The phosphorylation substrates for PKCα range from phospholamban to sarcomeric proteins such as troponin and titin, and phosphatases such as PP2A with ensuing effects on calcium handling and contractility^19^,^23^,^24^,^25^.

Intrigued by recent propositions that MLP may interact directly with PKCα^26^, and reports that MLP expression is down-regulated in failing mouse and human hearts^7^,^27^, we speculated that MLP may directly affect PKCα activity.

Our assays reveal that the presence of MLP inhibits autophosphorylation of PKCα as well as phosphorylation of downstream targets such as phospholamban. We demonstrate that in failing hearts PKCα is concentrated at the intercalated discs (ID) (specialized cell–cell contacts in cardiomyocytes) in a complex with the adaptor protein CARP1 and PLCβ1. In double knockout mice for CARP1 and MLP (CMP1), PKCα is no longer detected at the ID and the DCM phenotype does not develop. This is accompanied by normalized PKCα phosphorylation and expression levels. We propose a signalosome complex consisting of muscle ankyrin repeat proteins (MARPs) and PKCα that is regulated by MLP and whose persistent activation may play a role in chronic stress signalling in the failing heart.

## Results

### MLP directly inhibits PKCα activity

**Figure 1:**
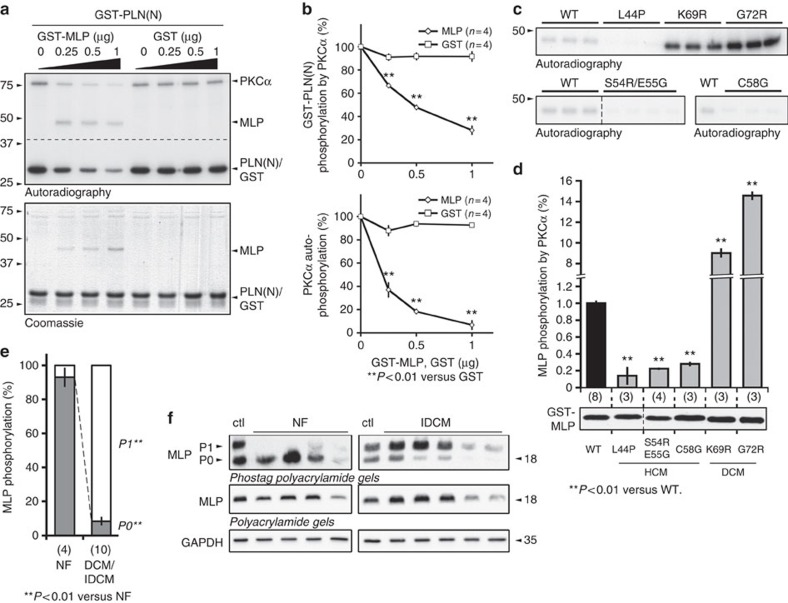
MLP is a substrate and inhibitor of PKCα kinase activity *in vitro*. (**a**) *In vitro* kinase assay using PKCα and GST tagged phospholamban cytoplasmic N-terminus (GST-PLN(N)) as substrate with increasing GST-MLP or GST amounts. Shown are representative autoradiography images for phosphorylated proteins (upper panel) or Coomassie staining for total proteins (lower panel). Dashed line indicates that proteins were run on the same gel, but imaged using different exposure times. (**b**) Quantification of GST-PLN(N) phosphorylation or PKCα auto-phosphorylation with increasing GST-MLP or GST concentrations. Mean values with s.e., sample size (*n*) and *P* values are shown. (**c**) *In vitro* kinase assay using PKCα and GST tagged WT and mutant MLP as substrate. Representative autoradiography images for either Leu44Pro (L44P), Lys69Arg (K69R), Gly72Arg (G72R) mutant MLP (top panel), or Ser54Arg/Glu55Gly (S54R/E55G) or Cys58Gly (C58G; bottom panel) mutant MLP (bottom panel) in comparison with WT MLP are shown. Dashed line indicates proteins were run on the same gel, but non-consecutive. (**d**) Quantification of MLP phosphorylation by PKCα. Phosphorylation efficiency of WT MLP was arbitrarily set to 1. Mutants identified in human HCM patients show lower MLP phosphorylation, while MLP mutants associated with DCM display increased phosphorylation values. A representative Coomassie stained gel showing total GST-tagged MLP used for *in vitro* kinase assays is depicted (bottom panel; dashed line indicates that proteins were run on the same gel, but non consecutive.). Shown are mean values and s.e., as well as *P* values and sample sizes (*n*, in brackets below bar graphs). (**e**,**f**) Analysis of MLP phosphorylation in IDCM. (**e**) Quantification of MLP phosphorylation indicates significantly elevated phosphorylation levels in IDCM patients, compared to NF controls. Shown are mean values and s.e., as well as sample size (*n*, in brackets below bar graphs), and *P* values. (**f**) MLP phosphorylation levels for quantification in (**e**) were determined by Phostag analysis. SDS samples of protein extracts of NF and IDCM patient heart samples were run on conventional SDS PAGE (middle) and on 12% polyacrylamide gels with 50 μM Phostag reagent (top) and immunoblotted for MLP. A human control sample with uncharacterized disease status (ctl) showing non-phosphorylated (P0) and phosphorylated (P1) MLP was used to indicate Phostag gel migration pattern. Phosphorylated proteins (P1) migrate slower due to their interaction with the Phostag reagent compared to unphosphorylated protein (P0). GAPDH (bottom) was used to show equal loading.

To investigate the possibility that MLP can directly modulate the activity of PKCα, we carried out *in vitro* phosphorylation assays using recombinantly expressed protein. Autoradiography showed that increasing amounts of MLP lead to decreased PKCα autophosphorylation and a decreased phosphorylation of the PKCα substrate phospholamban (Fig. 1a, for quantification see Fig. 1b). Furthermore, we found that MLP itself is a potential substrate of PKCα (Fig. 1a) and that gene mutations associated with human HCM result in reduced phosphorylation of MLP, while DCM-causing mutations show increased phosphorylation (Fig. 1c,d). Intriguingly, MLP phosphorylation appears to be significantly increased in heart samples of DCM patients (IDCM) as compared to non-failing (NF) controls (Fig. 1e,f). A similar shift in MLP phosphorylation was observed in Gα (q) overexpressing mice (Supplementary Fig. 1a,b), a mouse model for cardiac contractile failure^28^. A potential role of wild-type (WT) MLP in modulating PKCα signalling and activity in long-term heart failure may explain why the lack of MLP leads to DCM, and why all strategies that interfered with PKCα signalling prevented the development of a DCM phenotype in the MLP-deficient background^19^,^20^.

### PKCα concentrates at the ID in failing hearts

**Figure 2:**
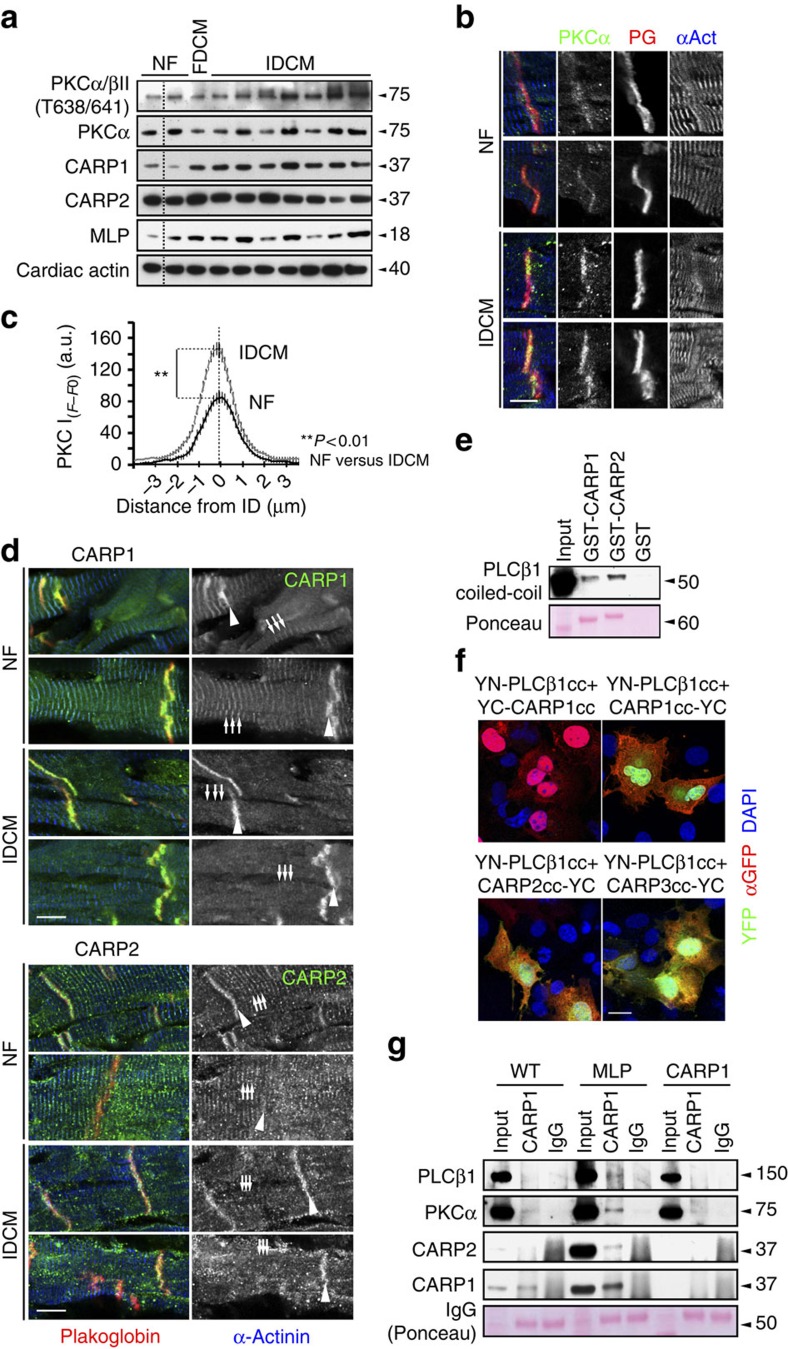
PKCα is sequestered in a multiprotein complex with CARP at the ID of failing hearts. (**a**) Analysis of PKCα phosphorylation levels (Thr638/641), PKCα, CARP1 and CARP2 protein levels in whole extracts of NF, familial DCM (FDCM) and IDCM patient heart samples. Cardiac actin was used as a loading control. Dotted lines indicate that samples were run on the same gel, but non-consecutive. (**b**) Immunofluorescence staining of human NF and IDCM heart sections using antibodies against PKCα (right, green). Plakoglobin and α-actinin were used as counterstains (red and blue in overlay, respectively). Scale bar=10 μm. (**c**) Quantification of immunofluorescence intensity (*F*−*F*_0_) over IDs from (**b**) shows that PKCα levels at the ID are increased in IDCM samples compared to NF controls. Sample sizes for analysed ID were *n*=24 from three NF heart samples and *n*=46 from five IDCM hearts. (**d**) Immunofluorescence staining of human NF and IDCM heart sections using antibodies against CARP1 (top, green in overlay) and CARP2 (bottom; green in overlay). The sarcomeric association (arrows) of CARP1 and CARP2 decreases in IDCM in favour of increased ID localization (arrowheads; for quantification see Supplementary Fig. 1d,e). Plakoglobin and α-actinin were used as counterstains (red and blue in overlay, respectively). Scale bar=10 μm. (**e**) Pull-down assay of GFP-PLCβ1 (phospholipase-C) with GST-CARP1, GST-CARP2 or GST indicated the coiled-coil domain within PLCβ1 as minimal binding site. Ponceau stain was used to visualize GST-CARP1 and GST-CARP2 protein levels, while equal amounts of GST (not shown) were used as a control. (**f**) Protein complementation assay using YFP (split-fluorescent protein assay) demonstrating antiparallel association (green in overlay) between coiled-coil domains of PLCβ1 tagged on the N-terminus with a YFP N-terminal fragment (YN) and CARP1 tagged on the C-terminus with YFP C-terminal fragment (YC; upper right panel). No association was seen when CARP1 was tagged on the N-terminus with YFP C-terminal fragment (upper left panel). Split-fluorescent protein assay also demonstrated antiparallel association (green in overlay) between coiled-coil domains of PLCβ1 tagged on the N-terminus with a YFP N-terminal fragment (YN) and either CARP2 (lower left panel) or CARP3 (lower right panel) tagged on the C-terminus with YFP C-terminal fragment (YC). Transfection efficiency was validated with a GFP antibody (red in the overlay), and DAPI (blue in overlay) was used as counterstain. Scale bar=20 μm. (**g**) Co-immunoprecipitation of PLCβ1, PKCα, CARP2 and CARP1 from soluble heart extracts of adult WT, MLP knockout or CARP1 knockout mice by using either CARP1 antibodies or normal rabbit IgG as control (visible in Ponceau stain).

While an increase in PKCα expression and activity is well established in the failing rodent heart^21^, the situation in humans is less clear^25^,^29^,^30^. Analysis of PKCα/β expression in heart samples from patients with idiopathic dilated cardiomyopathy (IDCM) by immunoblotting revealed a marked up-regulation of PKC phosphorylation at Thr638/641 in most patient samples (Fig. 2a). Confocal microscopy on immunostained heart sections from DCM patients showed a remarkable concentration of PKCα at the ID (Fig. 2b, quantification in 2c), which was not as evident in heart samples from NF controls. This supports published data that reported an increased signal for PKCα at the ID in human heart failure^29^. How does PKCα specifically target the ID? CARP1 is a stress marker that is consistently up-regulated in heart failure^12^,^31^ and a well-known scaffold protein (for reviews see refs 32, 33). Immunoblot data (Fig. 2a) showed increased expression of CARP1, but not the closely related CARP2/Ankrd2 in failing human heart samples. At the cellular level CARP1 is known to localize to the sarcomere and the nucleus, with increased nuclear presence following mechanical strain^34^. We observed no differences in nuclear versus cytoplasmic localization of CARP1 in either normal or DCM hearts (Supplementary Fig. 1c). However, in all functionally compromised hearts that we studied (MLP knockout and human DCM samples), CARP1 translocated from the sarcomere to the ID (Fig. 2d, for quantification see Supplementary Fig. 1d,e), whereas the localization of CARP2 was less affected (Fig. 2d, for quantification see Supplementary Fig. 1e). ID association for both proteins was recently also described by Jasnic-Savovic and colleagues^35^. Consistent with a previously reported interaction between CARP1 and phospholipase-C (PLC)^36^, we demonstrated in pull-down assays that the coiled coil region of PLCβ1, the main PLC isoform in hearts^37^, binds to both CARP1 and CARP2 (Fig. 2e). Protein complementation assays revealed that all CARP family members can interact with the coiled coil region of PLCβ1 and that this interaction happens in an antiparallel fashion (Fig. 2f). To investigate whether this complex also occurs in cardiac tissue *in situ*, we carried out immunoprecipitation experiments on cardiac extracts from MLP knockout hearts. CARP1 is exclusively expressed in cardiomyocytes and cannot be detected in fibroblasts (Supplementary Fig. 1f), therefore confirming that we specifically assayed the cardiomyocyte signalosome. Using CARP1 antibodies, we identified the presence of a signalosome complex in MLP knockout hearts containing CARP1, CARP2, PKCα and PLCβ1 (Fig. 2g). In contrast, these proteins were not pulled down from WT or CARP1 knockout hearts, adding support to our hypothesis that this complex only assembles in DCM/failing hearts. Using recombinant CARP1 or CARP2, we demonstrated that it is also possible to pull-down PLCβ1 and PKCα from MLP knockout hearts (Supplementary Fig. 1g). In left ventricle extracts from a subset of end-stage DCM patients the entire complex could be pulled down (Supplementary Fig. 1h).

These results indicate that CARP1 and CARP2 can sequester PKCα into a complex with PLCβ1. In the failing heart CARP1 is up-regulated and relocates from the sarcomere to the ID. This relocation and the ensuing retention of PKCα at the ID appear to be the crucial step in maladaptive signalling in failing hearts.

### PKC activity affects CARP and MLP expression

**Figure 3:**
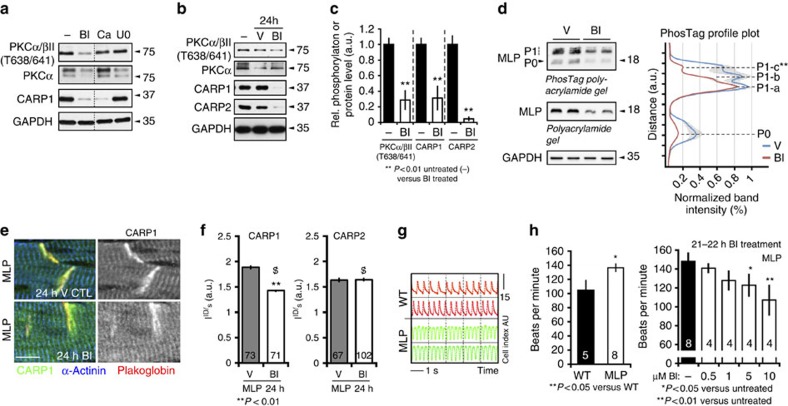
Inhibition of PKCα affects CARP1 expression and localization and decreases MLP phosphorylation. (**a**) Effect of PKC inhibitors (10 μM BI I-HCl; BI or 5 μM calphostin C; Ca) or MAP kinase kinase inhibitor (1 μM U0126; U0) treatment for 24 h on PKCα phosphorylation, PKCα and CARP1 expression levels in NMC compared to untreated controls (−). Dotted line indicates that samples were run on the same gel, but non-consecutive. GAPDH was used as loading control. (**b**) Immunoblot analysis of total cardiac samples from untreated (−), vehicle treated (DMSO, V) or BI treated adult MLP knockout mice (24 h after vehicle or BI-injection), blotted for phospho-PKCα (Thr638/641), PKCα, CARP1 or CARP2. GAPDH was used as a loading control. Shown are representative blots and quantification of changes for PKCα phosphorylation as well as CARP1 and CARP2 protein levels (**c**). Sample sizes for untreated controls and BI-treated animals were 4 and 3, respectively. *P* values are indicated in the figure. (**d**) BI treatment of NRC for 24 h leads to decreased MLP phosphorylation and protein level (see also Supplementary Fig. 1j,k). SDS samples of protein extracts from vehicle treated (V) or BI-treated NRC were run on conventional SDS PAGE (middle) and on 12% polyacrylamide gels containing 50 μM Phostag reagent (top) and immunoblotted for MLP. Phosphorylated MLP proteins (P1) migrate slower due to their interaction with the Phostag reagent, compared to unphosphorylated protein (P0). Phostag profile plot analysis of band intensities (right panel) revealed presence of three distinct P1 MLP phosphorylation bands (P1-a, P1-b and P1-c), which are putatively caused by several distinct phosphorylation sites in MLP. One of these phosphorylation sites (P1-c) is changed significantly upon BI treatment (red curve) compared to vehicle control (blue curve). Shown are normalized average band intensities and s.e. GAPDH was used as loading control (bottom). (**e**) Representative immunofluorescence images of adult heart sections from BI and vehicle treated MLP knockout mice stained with antibodies against CARP1 (green), α-actinin (blue) and plakoglobin (red). Scale bar=10 μm. (**f**) Analysis of fluorescence intensity ratios (I ^ID^/_S_) of CARP1 (left panel) and CARP2 stainings (right panel) between the ID region and the sarcomere (S) of either BI-treated or vehicle-treated 4-month-old MLP mice (see Supplementary Fig. 1d for methodology). Sample sizes (*n* in base of the bar; from two hearts per sample) and *P* values are indicated in the figure; $ denotes low expression of CARP1/CARP2 in BI treated hearts (as shown in Fig. 3b). (**g**,**h**) Label free impedance analysis of spontaneously contracting WT and MLP knockout NMC using the RTCA cardio system. Representative images of real-time beating activity of WT and MLP mouse cardiomyocytes (**g**) and quantification of their beating frequency (**h**). Effect of BI treatment on MLP cardiomyocyte beating frequencies (**h**, right panel). Bar graphs depict mean values and s.e. The number of independently measured wells (*n* in base of bar) and *P* values are indicated.

CARP1 is reportedly a target for many protein kinases including PKCα^38^, which we recently demonstrated through *in vitro* kinase assays^39^. Since CARP1 up-regulation and subcellular relocation appear to be crucial in setting up the pathological signalosome complex together with PKCα and PLC1β at the ID, we considered the possibility of an initial crosstalk between PKCα and CARP. Addition of kinase inhibitors to cultures of neonatal mouse cardiomyocytes (NMC) revealed that exposure to PKC inhibitors (bisindolylmaleimide (BI) or calphostin C (Ca)) not only reduced PKCα phosphorylation levels at Thr638/641, but also downregulated CARP1 expression levels (Fig. 3a). No effect was seen with a Map kinase kinase inhibitor (U0126; U0), again supporting the idea that the effect on CARP1 expression levels may be due to PKCα activity (Fig. 3a). The reduction in CARP1 expression following PKC inhibition could also be observed in cardiomyocytes cultured over several days as well as *in vivo*, when MLP knockout mice were peritoneally injected with BI (Fig. 3b,c, Supplementary Fig. 1i). The effect was not limited to CARP1, but also extended to CARP2 expression (Fig. 3b,c), which was significantly down-regulated in hearts of BI treated mice. More intriguingly, however, was the effect we observed on MLP phosphorylation and expression levels. PKC inhibition by BI decreased MLP expression levels, and more importantly MLP phosphorylation in cardiomyocytes (Fig. 3d). These data indicate that MLP is a direct phosphorylation target of PKC, and that this phosphorylation may affect MLP activity and protein levels (Supplementary Fig. 1k). In immunofluorescence-stained heart sections, PKC inhibition shifted CARP1 but not CARP2 localization from a more pronounced ID association to an increased sarcomeric signal (Fig. 3e, for quantification see 3f). Label-free measurements of NMC contractility demonstrated that inhibition of PKCα by BI normalized the higher beating frequencies usually observed in neonatal MLP knockout cardiomyocytes compared to WT controls (Fig. 3g,h).

These results show that elevated PKCα phosphorylation increases CARP expression levels. This is accompanied by the subcellular relocalization of both PKCα and CARP1 to the ID. The assembly of a signalosome consisting of CARP, PKCα and PLCβ1 at the ID leads to a stabilization of PKCα signalling activity. Inhibition of PKCα results in down-regulation of CARP expression, changes its subcellular targeting and probably leads to the disassembly of the signalosome. In addition, PKCα inhibition in WT cardiomyocytes leads to a decrease in MLP phosphorylation and expression.

We propose that acute signalling of this complex may be beneficial for the adaptation of the heart to stress^40^, while its chronic or pathological activation is maladaptive.

### Absent DCM phenotype in MLP CARP1/CARP2 double knockout mice

**Figure 4:**
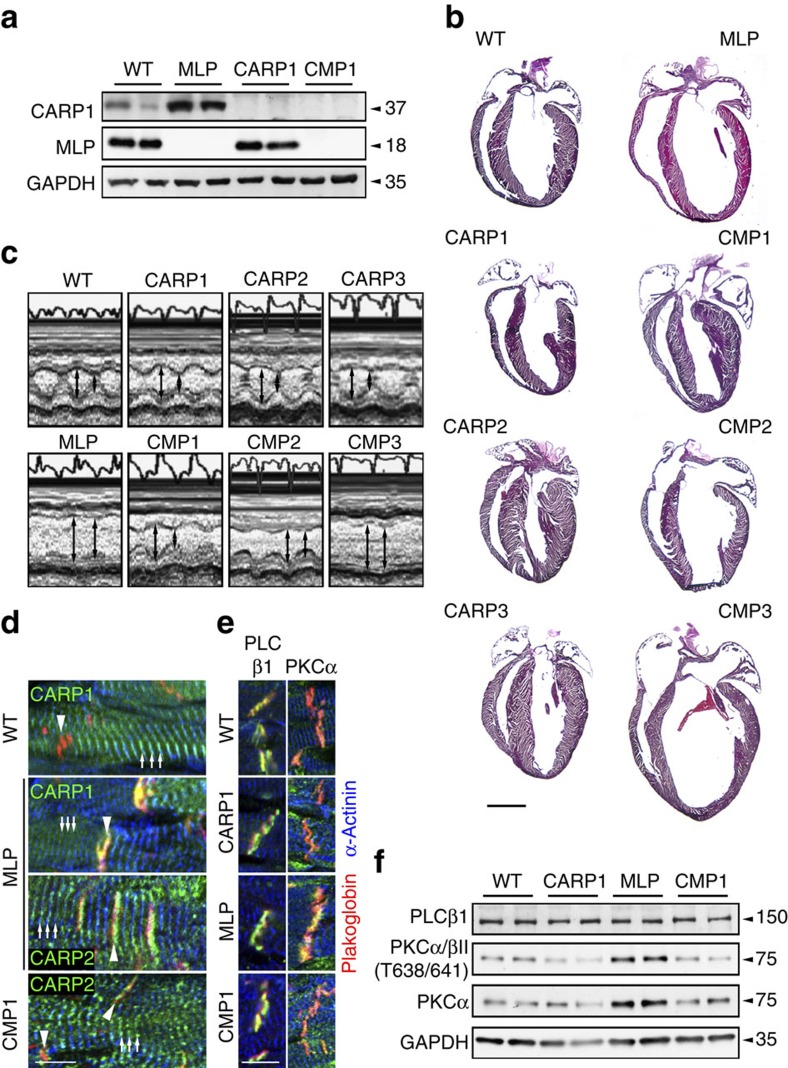
DCM in MLP knockout mice is prevented in the absence of CARP1 or CARP2. (**a**) Expression levels of CARP1, MLP and GAPDH as control in total heart extracts of WT, MLP knockout, CARP1 knockout and CARP1-MLP double knockout (CMP1) mice. (**b**) Cardiac morphology of WT, MLP, CARP1, CARP2 or CARP3 knockouts, as well as CARP1-MLP (CMP1), CARP2-MLP (CMP2) and CARP3-MLP (CMP3) double knockouts. Hematoxylin-eosin stained frozen heart sections are shown. Scale bar=2 mm. (**c**) Analysis of cardiac function using M-mode transthoracic echocardiography and representative M-mode images of hearts from WT, MLP, CARP1, CMP1, CARP2, CMP2, CARP3 and CMP3 animals. (**d**) Immunofluorescence staining of frozen heart sections from adult WT, MLP knockout and CMP1 double knockout animals stained with antibodies against CARP1 (green in overlay, top two panels) and CARP2 (green in overlay, bottom two panels). Arrowheads indicate ID and arrows show Z-disc localization, respectively. (**e**) Frozen sections of adult WT, CARP1, MLP knockout and CMP1 hearts stained with antibodies against PLCβ1 and PKCα (all green; see Supplementary Fig. 2h,i for quantification of PKCα localization at ID). (**d**,**e**) Plakoglobin and α-actinin were used as counterstains (red and blue in overlay, respectively). Scale bars=10 μm. (**f**) Immunoblot for PLCβ1, phospho-PKCα/βII (T638/641), and PKCα in WT, CARP1 knockout, MLP knockout and CMP1 double knockout total heart extracts. GAPDH was used as a loading control.

If CARP1 is indeed a crucial adaptor required to recruit PKCα stably to the ID, and to trigger pathological signalling in failing hearts, then its removal should prevent the development of heart failure in MLP knockout mice. Genetic ablation of any of the MARP family members in mice (CARP1/Ankrd1/MARP—encoded by the *Ankrd1* gene; CARP2/Ankrd2/ARPP encoded by the *Ankrd2* gene; CARP3/Ankrd23/DARP—encoded by the *Ankrd23* gene) had no effect on heart function at baseline, even in the case of a triple knockout^41^. To test our hypothesis, we crossed MLP knockout mice with the three MARP knockout lines and examined their cardiac phenotype. Immunoblot analysis of MLP and MARP expression revealed basal expression of CARP1, but no CARP2 in control hearts (Fig. 4a and Supplementary Fig. 2a,b). Hearts of MLP knockouts displayed increased expression as well as altered posttranslational modification of CARP1 and induced CARP2 expression (Fig. 4a, Supplementary Fig. 2a–c). CARP3 was not expressed in control hearts, and was not induced in MLP knockout hearts (Supplementary Fig. 2a). Histological and functional analyses revealed the previously reported phenotype of MLP knockout hearts (that is, dilation and heart failure^12^); in contrast double knockout mice for MLP and CARP1 (CMP1), and MLP and CARP2 (CMP2) had normal healthy hearts. Thus, the DCM phenotype does not develop in the absence of CARP1 or CARP2 (Fig. 4b,c, Supplementary Fig. 2d,e, Supplementary Tables 1 and 2). The hearts of double knockout mice for MLP and CARP3 (CMP3) were dilated and exhibited heart failure similar to the single MLP knockout mice (Fig. 4b,c, Supplementary Fig. 2d,e, Supplementary Table 3). CMP3 hearts had elevated expression levels of CARP1 and CARP2 as well as increased PKCα phosphorylation, suggesting activity of the same maladaptive signalling complex that we propose for MLP knockout mice (Supplementary Fig. 2a,f). The absence of a failing heart phenotype in the CMP1 animals was accompanied by redistribution of CARP2 to the sarcomeres (Fig. 4d, for quantification see Supplementary Fig. 2g). Most importantly PKCα no longer concentrated at the ID, while the PLCβ1 localization was indistinguishable between genotypes (Fig. 4e, Supplementary Fig. 2h,i). Analysis of PKCα phosphorylation levels revealed their normalization to control levels in the CMP1 heart compared to the MLP knockout heart (Fig. 4f). This suggests that it is the concentration of PKCα at the ID that triggers its pathological activity. Previously we characterized pathological changes at the cellular level in murine and human DCM samples such as elevated expression levels of N-RAP at the ID and re-expression of the foetal M-band marker EH-myomesin^7^,^42^,^43^. These changes were no longer seen in CMP1 or CMP2, but were still evident in CMP3 hearts (Supplementary Fig. 3a,b). However, while the lack of CARP1 or CARP2 expression prevents the development of a DCM phenotype, biomarkers for hypertrophy such as ANF, BNP and skeletal actin continued to be elevated at the mRNA level in CMP1 and CMP2 mice (Supplementary Fig. 4a–c). Also, double knockout hearts still showed some evidence of fibrosis (Supplementary Fig. 4d–f), although the amount was markedly reduced compared to MLP knockout mice.

In conclusion, we propose that the existence of a multiprotein complex consisting of CARP, PKCα and PLCβ1 at the ID of failing hearts is crucial for the maintenance of pathological signalling activity of PKCα. MLP can directly inhibit PKCα activity, and its absence is sufficient to lead to a DCM phenotype over time. Removal of CARP leads to the dissolution of the complex, where PKCα no longer concentrates at the ID, preventing the DCM phenotype in MLP knockout mice.

## Discussion

**Figure 5:**
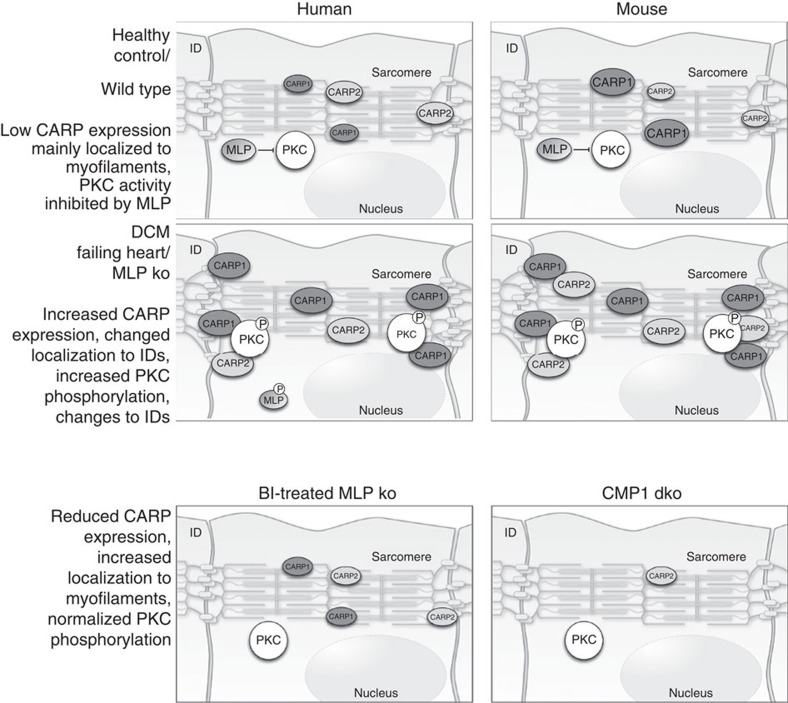
Model of signalling processes in functionally healthy and failing hearts. Schematic presentation of CARP1, CARP2, MLP and PKCα localizations, phosphorylation states and protein amounts (size of symbol) in the patient groups and analysed mouse models.

We demonstrate here the existence of a multiprotein complex composed of PKCα, PLC1β, CARP1 and CARP2 at the ID specifically in failing hearts. The retention of PKCα to this signalosome at the ID may lead to an amplification of signalling. The persistent shift in subcellular localization makes the difference between acute activation of PKCα that is beneficial for the heart to cope with transient stress, and chronic activation, which leads to detrimental downstream effects on calcium handling and contractile parameters. We propose that the up-regulation in expression and relocation of CARP1 or CARP2 from a sarcomeric localization to the ID is the crucial switch between acute and chronic PKCα activation (Fig. 5). We further show that MLP can directly inhibit PKCα activity *in vitro*, and demonstrate that removal of CARP by genetic means prevents the formation of this maladaptive signalling complex, thereby preventing the morphological, functional and molecular phenotype of DCM in MLP knockout mice.

These results explain at a molecular level, why previous strategies that prevented the development of a DCM phenotype in the MLP knockout mouse were successful^15^,^19^,^20^. Under normal circumstances MLP reduces excessive PKCα signalling in the heart. In MLP knockout mice this does no longer happen and DCM develops. Direct interference with the maladaptive PKCα signalling pathway by genetic or pharmacological strategies thus rescues the effect of the lack of MLP. The novel role of MLP as a PKCα inhibitor could also provide an explanation why overexpression of MLP does not have drastic effects on heart function under basal conditions^44^. Removal of phospholamban, which was found to rescue the MLP knockout phenotype^15^, leads to a reduction of free cytosolic calcium known to be required for PKCα activation.

We also noted that MLP itself is a substrate for PKCα activity. Indeed, DCM patient samples that show higher PKCα phosphorylation levels correlated with increased MLP phosphorylation levels compared to NF controls. Moreover we observed that DCM-causing MLP mutants were hyper-phosphorylated by PKCα *in vitro*, while MLP mutants that were associated with the development of HCM were hypo-phosphorylated. Inhibition of PKCα activity *in vitro* leads to decreased MLP phosphorylation and expression in cardiomyocytes. The exciting finding that the MLP phosphorylation state can be modulated by PKCα, correlates with different forms of cardiomyopathy and may affect its protein levels and/or activity again underlines the important role of this protein in cardiac stress signalling. At present it is unknown whether mutations in MLP or its various posttranslational modifications have an effect on oligomerization, which determines the subcellular localization and actin bundling activity of MLP^13^,^14^. Phostag blots of neonatal rat cardiomyocytes (NRC) indicate the presence of several distinct MLP phosphorylation sites, one of which was significantly changed upon PKC inhibition. It will be intriguing to investigate which posttranslational modifications of MLP indeed change its cell-biological properties. We propose that these posttranslational modifications of MLP are key to its biological function, and may ultimately determine where, when and how effectively it interferes with PKCα signalling.

CARP1 has for a long time been associated with mechanosignalling^34^ and was shown to be up-regulated in heart failure by several studies^12^,^31^,^45^. Recently its upregulated expression was shown to correlate directly with heart failure progression in IDCM^46^. So far most studies have proposed that nuclear signalling of CARP via ERK1/2 to molecules such as p53 and GATA4 is crucial for a hypertrophic response^47^. While there are conflicting results on the effect of CARP1 on ERK1/2 activation^40^,^47^, it appears to be increasingly recognized that stress sensing complexes associated with the titin I-band region, containing CARP1, or FHL1/2 and RAF/MEK/ERK may be partially responsible for mediating a hypertrophic response in hearts^48^. Our results now show that in heart failure CARP1 seems to be involved in a different signalling cascade at a distinct subcellular localization, namely boosting maladaptive PKCα signalling at the ID. Mutations in CARP1 were shown to cause both HCM and DCM^49^,^50^ and to display both gain of function and dominant negative effects on the contractile behaviour of engineered heart tissues^51^. We propose that whether mutant CARP1 leads to HCM or DCM may be correlated to differential effects on protein–protein interaction and in particular distinct activation of the two signalling pathways via ERK1/2 from the sarcomere to the nucleus (HCM) or via PKCα at the ID (DCM).

Our results demonstrate that one of the main differences between a healthy and a failing heart is the presence of PKCα at the ID. The analysis of PKCα/β expression levels in human hearts was inconclusive and it seems to be the change in subcellular targeting rather than mere expression levels that makes the crucial difference between a healthy and a failing heart. Why would active PKCα/β at the ID be so detrimental for the heart? Cell membranes are known to be the major site of PKC function (for review see ref. 52) and the signalosome complex that we describe here appears to be similar to previously described scaffold interactions that were shown to be crucial for PKC regulation^52^. Increased translocation of PKCα from the cytosolic to the membranous fraction marked the transition from pressure overload induced hypertrophy to congestive heart failure in guinea pigs^53^. In addition, constitutively active PKCα at the ID may interfere with another level of regulation in the challenged heart. β-adrenergic receptors are known to become insensitive following PKCα activation^54^ and adrenergic signalling is well known to play a role in heart disease^55^. Adrenergic receptors were previously shown to be concentrated at the ID in cardiomyocytes^56^. A failure to respond to fine-tuning by adrenergic signalling as shown by the altered contractile behaviour that we observed in cultured cardiomyocytes from MLP knockout mice, may over time lead to a failing heart phenotype.

Taken together, our results highlight novel roles for MLP and CARP in pathological signalling via their interactions with PKCα. This signalosome seems to be crucial for eliciting the maximal maladaptive response in the stressed heart, and CARP may be the proposed missing link^57^. Modulation of this complex may offer new therapeutic options to prevent heart failure.

## Methods

### Bacterial protein expression and *in vitro* kinase assay

Expression of GST-fusion proteins was done using BL21 cells (C601003; Invitrogen; Life Technologies, Carlsbad, CA). BL21 cells were transformed with GST-expression vectors (GST-C1, GST-CARP1, GST-CARP2, GST-MLP (WT and mutants), GST-PLN(N)) and grown in 400 ml LB-medium supplemented with 50 μg ml^−1^ carbenicillin on an orbital shaker at 37 °C to an OD600 of >1. After incubation on ice for 15 min, expression of GST or GST-fusion proteins was induced by addition of 0.2 mM IPTG (Isopropyl β-D-1-thiogalactopyranoside) and production of proteins was allowed to proceed over night at 18 °C. Cells were collected by centrifugation and lysed into 40 ml ice cold lysis buffer (150 mM NaCl, 10 mM Tris-HCl pH 8, 1% Triton X100), followed by sonication for 1 min at 4 °C at 70% output (Vibracell, Sonics & Materials Inc., Newtown, CT). Removal of insoluble cell debris was done by centrifugation (45 min at 11,000 r.p.m., 4 °C; Sorvall) and the supernatant was incubated with 400 μl glutathione-sepharose beads (Pharmacia) for 2 h at 4 °C with agitation. After washing of beads with ice cold PBS for four times, bound protein was eluted with GST-elution buffer (150 mM NaCl, 50 mM Tris-HCl pH 7.4, 150 mM reduced glutathione) and dialyzed over night at 4 °C against dialysis buffer (20 mM HEPES pH7.4, 10 mM MgSO_4_, 0.1 mM CaCl_2_). Measurement of protein concentration was done using the Bradford assay (BioRad) or through densitometry of Coomassie stained SDS-PAGE gels. Proteins were frozen in liquid nitrogen and stored at −80 °C until further use in *in vitro* kinase assays or for GST pull-down assays.

For *in vitro* kinase assays 2 μg of GST-PLN(N) or GST-MLP (WT or mutant) were combined with purified PKCα (approximately 10 ng/reaction, P0329, Sigma-Aldrich) in kinase buffer (20 mM HEPES KOH pH7.4, 10 mM MgSO4, 0.1 mM CaCl2) supplemented with either radioactively labelled [gamma-32P]ATP (Amersham Biosciences) or unlabelled ATP (20 mM) and increasing amounts (0.25 μg, 0.5 μg or 1 μg) of either GST-MLP or GST. The reaction mix was supplied with a combination of 1 μg μl^−1^ phosphatidylserine (1,2-diacyl-sn-glycero-3-phospho-L-serine) and 2 μg μl^−1^ diacylglycerol (DAG; 1,2-Dioleoyl-sn-glycerol) dissolved in resuspension buffer (10 mM HEPES pH 7.4, 0.3% Triton X-100). Following incubation at 30 °C for 30 min, samples were mixed with SDS-sample buffer (BioRad) and separated using SDS-PAGE. For radioactively labelled proteins, gels were coomassie-stained or fixed, dried on a BioRad gel dryer and analysed using X-ray films and autoradiography^39^. Densitometric quantification of phosphorylation levels was done by scanning X-ray films followed by measurement of band intensity using ImageJ (NIH).

### Human tissues

Human specimens were obtained from the Sydney Heart Bank at the University of Sydney and signed patient consent was obtained for all samples in this tissue bank. All failing samples were collected in the heart transplant theatres at St Vincent's Hospital from patients in end-stage heart failure and were frozen in liquid nitrogen within 15–20 min of the loss of coronary artery flow (cross-clamp). Human Research Ethics Committee approval was obtained by St Vincent's Hospital (#H03/118), and by the University of Sydney Human Research Ethics Committee (#12146, #159401). Human tissue was used in accordance with the ethical guidelines of King's College London (College Research Ethical Committee 04/05–74; REC reference 12/EM/0106), under current UK law. For protein, histological and immunofluorescence analysis, samples from the left ventricular free walls of patients in end-stage heart failure with familial (FDCM) or IDCM were used. NF donor hearts, which were cardiopleged, but not required for heart transplantation, were provided by the Australian Red Cross Blood Transfusion Service. Typically these hearts were not transplanted because of tissue incompatibility.

### Generation of constructs

Bacterial and eukaryotic expression constructs for human MLP (HSU49837), the cytoplasmic domain of mouse phospholamban (PLN(N), amino acids 1–34; NM_023129), human CARP1/Ankrd1 (NM_014391), human CARP2/Ankrd2 (NM_020349), human CARP3/Ankrd23 (NM_144994), human phospholipase C β1 coiled-coil domains (NM_015192, amino acids 978–1138) were done by amplifying the coding sequence via PCR from a human cardiac cDNA library (Clontech) or from mouse cardiac cDNA and subsequent in-frame cloning into the pEGFP-C1 (Clontech), pEGFP-N1 (Clontech), HA-N1 (a modified version of the pEGFP-N1 vector that replaces the EGFP coding sequence with the HA-tag), or pGST-C1 vector (a modified version of the pGEX-2T bacterial expression vector; Amersham Pharmacia^39^). Generation of bacterial GST-tagged mutant human MLP expression constructs was done as described previously^11^.

Generation of protein complementation constructs was done through in-frame ligation of the coiled-coil region of CARP1 (amino acids 1–122), the coiled-coil region of CARP2 (amino acids 1–146), the coiled-coil region of CARP3 (amino acids 1–109), and the coiled-coil region of human phospholipase C β1 (PLCβ1; amino acids 980–1140) into the protein complementation vectors (split-YFP) YN-C1, YC-C1 or YC-N1 (ref. 39). This effectively generates either N-terminally tagged fusion proteins with the N-terminal (YN) or C-terminal (YC) half of YFP, or C-terminally tagged fusion proteins with the C-terminal half of YFP.

All constructs were verified for in-frame integration and correct sequence by sequencing.

### Protein expression analysis

Total protein extracts of hearts for immunoblot or biochemical analysis were generated by homogenizing ventricular samples either directly into SDS-sample buffer (BioRad) or ice-cold IP-buffer (150 mM NaCl, Tris-HCl pH 8, 1 mM DTT, 1 × Complete Protease Inhibitor EDTA-free (Roche), 1 × PhosSTOP (Roche), 0.2% NP-40, 0.2% SDS) by using a polytron blade homogenizer (Pro Scientific Inc). Total proteins from NMC, neonatal cardiac fibroblasts or transfected COS-1 cell cultures were extracted by washing cells with PBS at room temperature and solubilization of cells directly into SDS-sample buffer or ice-cold IP-buffer using a cell scraper. Protein extracts were immediately transferred into Eppendorf tubes and stored on ice for immediate use or snap-frozen into liquid nitrogen and stored at −20 °C or −80 °C. Protein amounts were normalized by densitometry of Coomassie stained SDS PAGE.

Normalized total protein extracts were run on uniform 15% acrylamide, 10% acrylamide, or 4–20% acrylamide gradient SDS-PAGE gels (BioRad, Invitrogen), followed by immunoblotting on nitrocellulose membranes (BioRad) using wet blot technology. The nitrocellulose membranes were stained with Ponceau Red, blocked with 5% non fat dry milk (Sainsbury's) or 3% Bovine Serum Albumin (Sigma) in Low Salt Buffer (0.9% NaCl, 9 mM Tris pH 7.4, 0.1% Tween-20) and sequential incubation with the appropriate primary and secondary antibodies with intermittent washing in Low Salt Buffer was performed. Results from the chemiluminescence reaction were visualized on Fuji medical X-ray films. For 2D gel analysis, protein samples were diluted 1:1 into IEF sample buffer (Life Technologies), and first dimension was run on IEF pH3–10 gels (Life Technologies), followed by 12% acrylamide SDS-PAGE according to the manufacturer's instructions.

### Phostag gels

For analysis of MLP phosphorylation levels, 20 mg of frozen cardiac tissue samples were homogenized into 200 μl freshly prepared ice-cold TLB lysis buffer (20 mM Tris HCL pH 7.6, 138 mM NaCl, 5% glycerol, 1% Triton, 5 mM DTT, 0.5 mM Sodium ortho-vanadate, Mini Protease inhibitor tablet (no EDTA; Roche), Phos-stop tablet (Roche)). Samples were briefly sonicated, incubated on ice for 20 min, centrifuged at 4 °C at 14,000 r.p.m. for 15 min, and supernatants were used for further analysis via Phostag gel analysis^58^ after determination of protein concentrations. Protein samples with normalized concentrations were supplemented with SDS sample buffer, boiled for 5 min, and either run on a 12% poly-acrylamide SDS-PAGE, or a 12% poly-acrylamide SDS PAGE supplemented with 50 μM Manganese Phostag (WAKO Chemicals). After end of electrophoresis, gels were washed once in immunoblot transfer buffer supplemented with 10 mM EDTA for 10 min, followed by a wash in immunoblot transfer buffer for another 10 min, and prepared for immunoblot transfer and subsequent detection and analysis following standard procedures.

### Co-immunoprecipitation and GST pull-down assays

For biochemical protein–protein interaction assays, protein extracts in IP-buffer were sonicated for 1 min at 30% output on ice (Vibracell, Sonics & Materials Inc.), followed by centrifugation at 14,000 r.p.m. (4 °C) for 10 min to separate insoluble proteins. Soluble proteins were either incubated with 1 μg of primary antibody or control serum for 2 h, or incubated with 2 μg of GST or GST-fusion protein at 4 °C. Following immuno-complex formation in co-immunoprecipitation assays, protein extracts were incubated with protein G-linked magnetic beads (Dynabeads; Invitrogen) for an additional hour at 4 °C with agitation. For GST pull-down assays, protein extracts were incubated with glutathione-sepharose 4B resin (Pharmacia) for an additional 2 h at 4 °C with agitation. After incubation, beads were washed three times with ice-cold PBS, resuspended in sample buffer (BioRad), and analysed by SDS PAGE, followed by immunoblotting on nitrocellulose membranes^39^.

### Animals

All procedures were reviewed and approved by the Animal Care and Use Committee at the University of California San Diego. MLP and MARP knockout mice as well as Gα (q) overexpressing mice were previously described^12^,^28^,^41^. All animals were in a mixed sv129/black swiss background. For physiological experiments only male mice were used, samples used for immunohistology and immunoblotting were from both genders. Unless stated otherwise, animals in the age range between 4 and 8 months were used for the experiments. Oligonucleotides used for genotyping via PCR analysis of tail DNA can be found in Supplementary Table 4.

The mice were fed ad libitum with a standard diet and maintained in a temperature and light-controlled room (22 °C, 14 h light/10 h dark). Treatment of MLP knockout animals with PKC inhibitor was done similar to a previously described method^20^. In short, 4-months old male MLP mice were injected subcutaneous with a single dose of either PKC inhibitor BI I-HCl (BI; sc-24004, Santa Cruz Biotechnology, 1 μg g^−1^ body weight) or DMSO (vehicle). Ventricular heart tissue of mice was dissected after 24 h following the injection and snap frozen in liquid nitrogen for further analysis, or processed for immunofluorescence analysis.

The protocol for animal handling and treatment procedures was in accordance with the guidelines of the Laboratory Animal Services at the University of California, San Diego and guidelines presented in the National Research Council's (NCR) ‘Guide for Care and Use of Laboratory Animals' published by the Institute for Laboratory Animal Research of the National Academy of Science, Bethesda, MD, 2011.

For echocardiography analysis 2-, 4-, 6- and 12-month-old male control, MLP, MARP and MLP-MARP double knockout mice were analysed. For protein, mRNA, histological, immunofluorescence and morphological analysis, the left ventricular free wall of hearts from 3–9-month-old adult male and female mice were used. Investigation of gross cardiac morphology by histology used whole hearts that included ventricles and atria.

### Transthoracic echocardiography

Adult male mice at 2, 4, 6 and 12 months of age were analysed as previously described^41^. Briefly, mice were anesthetized with 1% isoflurane and cardiac function was measured with a Philips Sonos 5500 machine (Philips Medical Systems, Andover, MA) equipped with a 15-MHz transducer. M-mode tracings of semi-conscious mice were recorded and analysed for left ventricular posterior wall and inter-ventricular septal thickness, as well as left ventricular chamber dimensions (LVID) at both end systole and end diastole. Heart contractility, shown as fractional shortening (%FS) was calculated as previously described^41^.

### Antibodies

The following primary antibodies were used for biochemical analysis of protein and phosphorylation levels (dilutions given in brackets are for immunoblotting, the antibodies were used 10 times more concentrated for immunohistology): GAPDH (clone 6C5; Santa Cruz Biotechnology; 1:1000), cardiac Actin (clone Ac1–20.4.2, Progen Biotechnik; 1:2000), collagen-1 (ab34710, Abcam; 1:1000), smooth-muscle actin (clone 1A4, DAKO: 1:1000), CD-31/PECAM-1 (550274, BD Pharmingen; 1:5000), α-sarcomeric actinin (clone EA53, A7811, Sigma-Aldrich; 1:5000), PLCβ1 (sc-205, Santa Cruz Biotechnology; 1:1000), PKCα (sc-208, Santa Cruz Biotechnology; #2056, Cell Signalling; 1:1000), phospho-PKCα (T638/641 #9375, Cell Signalling; 1:1000), plakoglobin/γ-catenin (SAB2500802, Sigma-Aldrich; 1:1000), sarcomeric myosin-heavy chain (A4.1025, Developmental Studies Hybridoma Bank, University of Iowa; 1:100), PDGF-Receptor α (AF1062, R&D; 1:1000), GFP-tag (11814460001 Roche; 1:1000). Normal rabbit IgG was from Santa Cruz Biotechnology (sc-2027), while normal donkey serum was from Jackson ImmunoResearch.

Polyclonal antibodies against MARP proteins (used 1:1000) were either produced in house or a kind gift of Dr Siegfried Labeit (University of Heidelberg) and produced by Myomedix. Monoclonal antibodies against myomesin (used 1:200) and MLP (used 1:1000) were described previously^7^,^11^. Polyclonal antibodies against EH-myomesin (used 1:500 for immunofluorescence) were generated by Dr Irina Agarkova^42^. Polyclonal antibodies against N-RAP (used 1:100 for immunofluorescence) were generated by BioScience (Göttingen, Germany).

All fluorescently or enzymatically linked secondary antibodies were either from DAKO, Jackson ImmunoResearch or GE Healthcare Life Sciences and used at 1:100 (fluorescent) or 1:1000 (enzymatic) dilutions.

### Quantitative real-time PCR analysis (qPCR)

Ventricular samples were homogenized directly into Trizol reagent (Invitrogen) to extract total RNA according to the manufacturers instructions. First strand cDNA was generated from 2 μg of total mRNA using random hexamers and Superscript II reverse transcriptase. Oligonucleotides optimized for qPCR (Supplementary Table 5) of murine ANF, BNP, skeletal actin, CARP1 and GAPDH were used in reactions employing the PerfeCTa SYBR green real-time PCR mix (Quanta BioSciences) and a CFX96 thermocycler (BioRad). Samples were normalized to GAPDH. If not stated otherwise, three biological replicates were analysed per sample group.

### Cell culture

The isolation of NMC and NRC was performed using collagenase/dispase digestion, respectively^59^. Hearts from neonatal rats and mice were initially dissected in PBS containing 20 mM BDM (2,3-butanedione monoxime; Sigma) and minced. Subsequently they were subjected to digestion with collagenase/dispase (Roche; 15 mg in 10 ml L15 medium with 20 mM BDM) for 3 × 20 min at 37 °C with gentle agitation. Alternatively, cardiomyocytes were isolated using the Neonatal Cardiomyocyte isolation kit (Miltenyi Biotec) according to the manufacturer's instructions. Individual cells were released by trituration with a 10 ml tissue culture pipette and undigested tissue pieces were removed using a cell strainer (Falcon). After centrifugation at 80 g, the pellet of isolated cells was resuspended in Plating Medium (68% DMEM, 17% Medium M199, 10% horse serum, 5% foetal calf serum, 4 mM glutamine, and 1% penicillin-streptomycin, all from Sigma), plated into collagen-coated plastic dishes and incubated over night. The next day, the cells were switched to Maintenance Medium (20% Medium M199, 75% DBSS-K, 4% horse serum, 4 mM glutamine, 1% penicillin/streptomycin, 0.1 mM phenylephrine; DBSS-K: 6.8 g l^−1^ NaCl, 0.14 mM NaH2PO4, 0.2 mM CaCl_2_, 0.2 mM MgSO4, 1 mM dextrose, 2.7 mM NaHCO3). Treatment of NMC and NRC using BI I-HCl (BI; sc-24004, Santa Cruz Biotechnology, 10 μM), calphostin C (Ca; sc-3545, Santa Cruz Biotechnology, 5 μM) or U0126 (U0; sc-222395, Santa Cruz Biotechnology, 1 μM) was done by supplementing the maintenance medium for 24 h with inhibitors or vehicle, followed by biochemical analysis of protein extracts.

For analysis of CARP1 and MLP expression in cardiomyocyte versus cardiac fibroblasts, cardiac fibroblasts were separated during the NMC isolation procedure, and cultured for an additional 2 days in DMEM (high glucose), supplemented with 10% foetal calf serum and 1% penicillin/streptomycin medium.

COS-1 cells were cultured in DMEM with 10% foetal calf serum and transfected mixing 1 μg of plasmid DNA and 3 μl of Lipofectamine-2000 (Life Technologies Carlsbad, CA) in 300 μl DMEM and adding the mix to the cells.

### Immunofluorescence and histology

Immunostaining of frozen sections on glass-slides (Colorfrost Plus, Thermo Scientific) and cells in cell-culture dishes (Nunc) was done as described previously^7^,^39^. In brief, immunostaining of frozen heart sections was achieved by fixing 12 μm sections (Leica CM1850 cryostat) in ice-cold acetone for 5 min at −20 °C, followed by rehydration of the tissue section with PBS at room temperature for 5 min. Cultured NMC or COS-1 cells were fixed with 4% paraformaldehyde solution in PBS for 5 min at 37 °C, followed by a wash with PBS at room temperature. After permeabilization using 0.2% triton-X100 in PBS for 5 min, sections or cells were incubated with primary antibodies in gold buffer (GB; 20 mM Tris-HCl, pH 7.5, 155 mM NaCl, 2 mM ethylene glycol tetraacetic acid, 2 mM MgCl_2_, 1% bovine serum albumin; for antibody dilutions see Antibodies paragraph) supplemented with 5% normal donkey serum over night at 4 °C in a humid chamber. After washing of sections or cells for three times with PBS to remove unbound primary antibodies, sections or cells were incubated with secondary antibodies in GB supplemented with 5% normal donkey serum and 2 mg ml^−1^ 4′,6-diamidino-2-phenylindole (10 μg ml^−1^ DAPI, Sigma-Aldrich; for antibody dilutions see Antibodies paragraph) at room temperature in a humid chamber. Following washing of sections or cells with PBS (three times) for 5 min at room temperature, sections or cells were embedded in fluorescent mounting medium (DAKO) and mounted with coverslips.

For hematoxylin-eosin staining, 20 μm frozen sections were fixed for 5 min at room temperature using a 4% paraformaldehyde solution in PBS. Following fixation, sections were washed in water, incubated in Weigert's Hematoxylin solution (HT1079, Sigma-Aldrich) for 5 min, briefly rinsed with water and counterstained with Eosin-Y solution (Richard Allan Sci.) for 2 min. After dehydration of sections using increasing percentages of alcohol (50% for 2 min, 75% for 2 min, 95% for 2 min, twice 100% for 5 min), samples were washed twice in Histo-Clear (National Diagnostics) for 10 min and mounted using Permount (Fisher Sci.).

### Confocal microscopy

Fluorescently stained samples were imaged using an Olympus Fluoview 1000 confocal microscope equipped with 40 × oil immersion lens, or a LEICA TCS-SP5 confocal microscope equipped with a 63 × glycerol immersion objective in sequential scanning mode and zoom rates between one and three. Histology samples were recorded by a SPOT camera and imaging software, using a Nikon epifluorescence microscope equipped with a 5x air objective in bright-field mode.

### Image processing and statistical analysis

Images were processed using ImageJ (NIH) equipped with the LOCI bio-formats plugin and Photoshop (Adobe). Statistical analysis was done using Excel (Microsoft). Data presented are mean values±s.e. Significance was evaluated by the two-tailed student's *t*-test. Sample size (*n*-values) and *P* values are indicated in the figures and/or figure legends.

For analysis of PKC localization over ID (Fig. 2c, Supplementary Fig. 2h), profile plots were measured over aligned ID using the ImageJ plugin ‘RGB profile plot'. Plot values (PKC fluorescence intensity and plakoglobin fluorescence intensity) were exported into Excel. The plakoglobin profile was used to establish the precise localization of the ID. The corresponding PKC intensity (*F*) at the ID was subtracted with the background PKC intensity (F0) to generate the background corrected profile PKCα plots (*F*−*F*0). Profile plots were centred at the ID, and the average profile plot and s.e. were calculated for each group, and used to generate the figure. Data presented in Fig. 2c and Supplementary Fig. 2h are mean values±s.e. Significance was evaluated by the two-tailed student's *t*-test over fluorescence intensity values (*F*−*F*0) at the ID. Sample size (*n*-values) and *P* values are indicated in the figures and/or figure legends.

For Phostag profile plot analysis (Fig. 3d), MLP bands were analysed using ImageJ and the ‘Plot Profile' tool. Data were exported to Excel, aligned and normalized, and the resulting average plot profile for each group, including s.e. were used to generate the figure. Significance was evaluated by student's *t*-test analysis.

For analysis of CARP1, CARP2 and α-Actinin fluorescence intensity ratios (I^ID^/_S_) between the ID and the sarcomere (S), identical-sized boxes encircling a portion of the ID, and an adjacent sarcomere were selected, and RGB values were measured in ImageJ using the ‘RGB measure' plugin. After measurement of fluorescence intensities, individual and average intensity ratios as well as s.e. and significance was calculated in Excel. Data presented in Fig. 3f, Supplementary Figs 1e and 2g are mean values±s.e. Significance was evaluated by the two-tailed student's *t*-test. Sample size (*n*-values) and *P* values are indicated in the figures and/or figure legends.

### Label-free measurement of cardiomyocyte contraction

Live label-free impedance measurements (cell index) of cardiomyocyte beating frequency (beats per minute) and contraction behaviour were done using the xCelligence RTCA cardio system (ACEA Biosciences)^60^. Basal contractile behaviour of cells was analysed two days after plating of NMCs into 96 well plates. The effect of increasing concentrations (0.5, 1, 5 and 10 μM) of the PKC inhibitor BI I-HCl (BI) in comparison to untreated cells was evaluated 21–22 h after addition of the small molecule inhibitor to the maintenance culture medium.

### Data availability

All relevant data are available from the authors.

## Additional information

**How to cite this article:** Lange, S. *et al*. MLP and CARP are linked to chronic PKCα signalling in dilated cardiomyopathy. *Nat. Commun.* 7:12120 doi: 10.1038/ncomms12120 (2016).

## Supplementary Material

Supplementary Information Supplementary Figures 1-4 and Supplementary Tables 1-5
