# Supplementary material for: MLP and CARP are linked to chronic PKCα signalling in dilated cardiomyopathy
Source: Nat Commun. 2016 Jun 29;7:12120. doi: 10.1038/ncomms12120 (PMC4931343; doi:10.1038/ncomms12120)
Supplement: Supplementary Information — Supplementary Figures 1-4 and Supplementary Tables 1-5 [file ncomms12120-s1.pdf]

Supplementary Figures and Tables

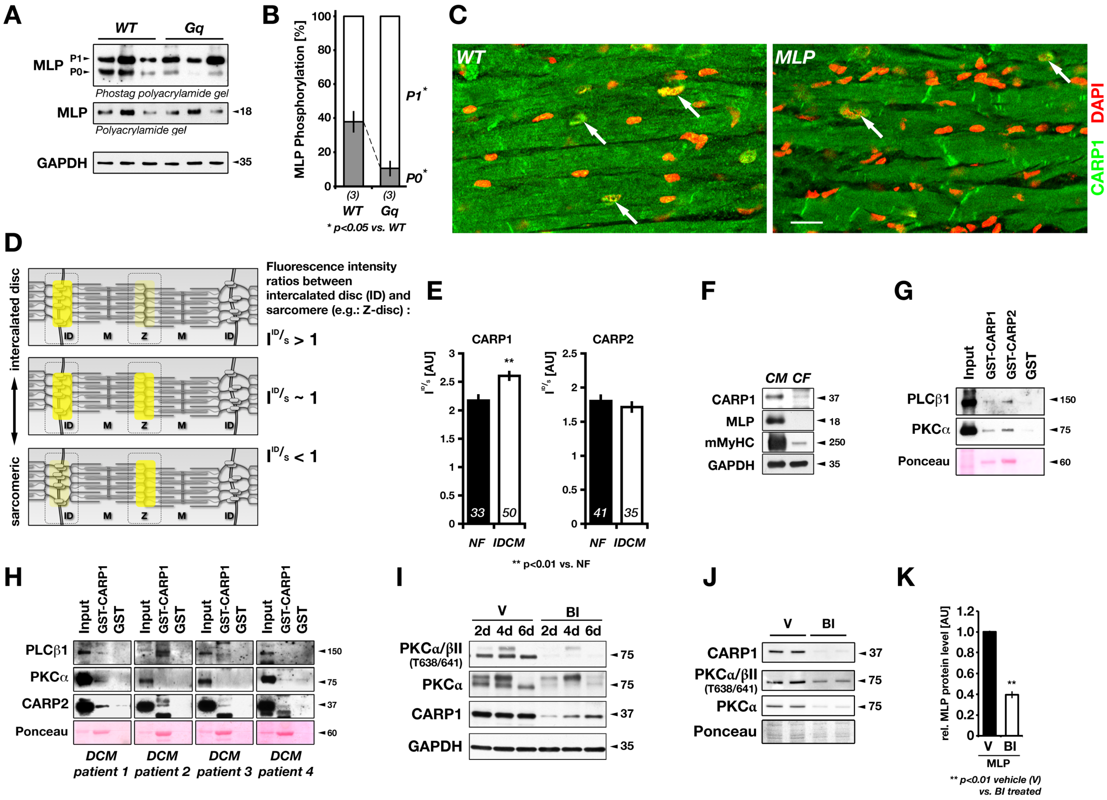

Supplementary Figure 1: (A, B) Analysis of MLP phosphorylation in Gα (q) mouse heart samples. (A) MLP phosphorylation levels for quantification in (B) were determined by Phostag analysis. SDS samples of protein extracts of wildtype and Gα (q) heart samples were run on conventional SDS PAGE (middle) and on 12% polyacrylamide gels with 50 μM Phostag reagent (top) and immunoblotted for MLP. Phosphorylated proteins (P1) migrate slower due to their interaction with the Phostag reagent compared to unphosphorylated protein (P0). GAPDH (bottom) was used to show equal loading. (B) Quantification MLP phosphorylation indicates significantly elevated phosphorylation levels in Gα (q) mouse hearts, compared to wildtype controls (WT). Shown are mean-values and standard errors, as well as sample size (n, in brackets below bar graphs), and p-values. (C) Immunofluorescence staining of frozen heart sections from adult wildtype (WT) and MLP knockout animals stained with antibodies against CARP1 (in green). DAPI (in red) was used as nuclear counterstain. Arrows indicate nuclear CARP localization, which is observable independent of cardiac phenotype. Scale bar =20μm. (D) Schematic for the analysis of fluorescence (yellow boxes) intensity ratios (I $^{ID}/_{S}$) between the intercalated disk (ID) and the sarcomere (S), and interpretation of results. Please see Materials and Methods section for a detailed description of the methodology. (E) Analysis of fluorescence intensity ratios of CARP1, and CARP2 stainings between the intercalated disks (ID) and the sarcomeres (S) of heart sections from non failing (NF) and idiopathic DCM (IDCM) patients. Displayed are mean values, standard errors, sample size (n in base of graph; two heart samples per group) and p-values. (F) Analysis of CARP1 and MLP protein levels in cardiomyocytes (CM) and cardiac fibroblasts (CF). Muscle specific myosin heavy chain (mMyHC; clone A4.1025) was used as cardiac control and GAPDH as loading control. (G) Pulldown assay of phospholipase-C (PLC) β1 and PKCα from MLP knockout hearts with either GST-CARP1, GST-CARP2 or GST. (H) Pulldown assay of phospholipase-C (PLC)β1, PKCα and CARP2 from soluble extracts of DCM patient hearts with either GST-

CARP1 or GST. Note that only a subset of patients (patient 1) display positive interactions of all investigated proteins with GST-CARP1. (G, H) Ponceau stains were used to visualize equal loading of GST proteins (GST control not shown). (I) Immunoblot analysis of total protein samples of neonatal mouse cardiomyocytes treated for 2, 4 or 6 days with 10 μM bisindolylmaleimide-I HCl (BI) and vehicle-treated controls (DMSO, V), stained with antibodies against phospho-PKCα (Thr638/641), PKCα or CARP1. GAPDH was used as loading control. (J, K) BI treatment of neonatal rat cardiomyocytes. (J) Immunoblot analysis of total protein samples of neonatal rat cardiomyocytes treated for 24 hours with 10 μM bisindolylmaleimide-I HCl (BI) and vehicle-treated controls (DMSO, V), stained with antibodies against CARP1, phospho-PKCα (Thr638/641) or PKCα. Ponceau Red staining is shown as a loading control. (K) Quantification of MLP expression levels after 24 hour BI treatment of neonatal rat cardiomyocytes (in triplicate), compared to vehicle treated controls (see Figure 3D).

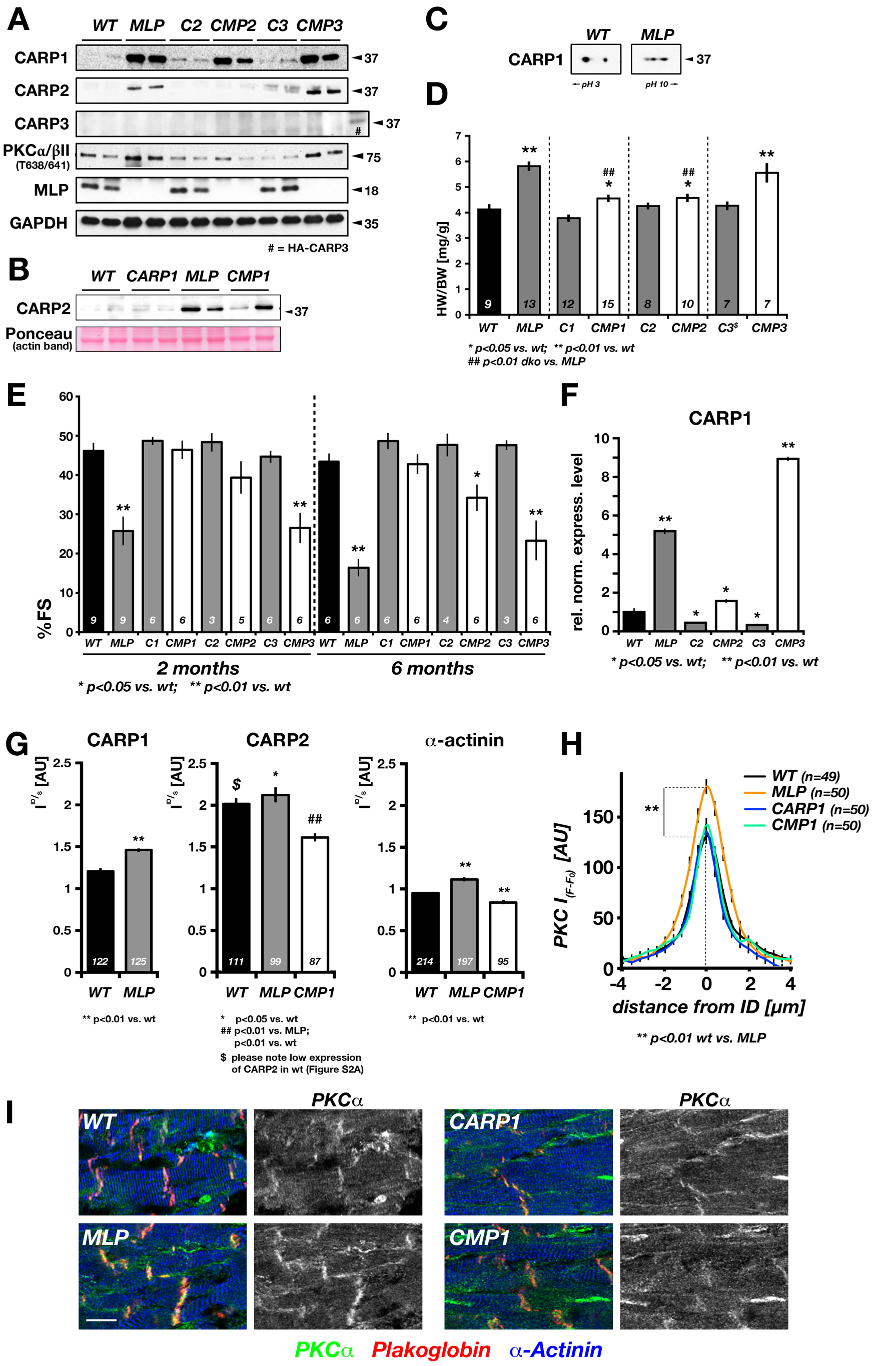

Supplementary Figure 2: (A) Measurement of CARP1, CARP2, CARP3 and MLP protein expression levels (left panel) in total cardiac extracts of WT, MLP knockout (MLP), CARP2 knockout (C2), CMP2 double knockout, CARP3 knockout (C3) and CMP3 double knockout mice. HA-CARP3 in the CARP3 panel was used as detection control (pound sign), and GAPDH was used as loading control. (B) CARP2 expression levels in whole heart extracts from WT, CARP1 knockout, MLP knockout and CMP1 double knockouts. Ponceau stained actin band was used as loading control. (C) Identification of differential CARP1 posttranslational modification using 2D gel analysis of wildtype and MLP whole heart extracts. (D) Heart-weight body-weight ratios of adult (4-6 months; 4-7 months for CARP3 knockout) WT, MLP knockout, CARP1 knockout (C1), CMP1 double knockout, CARP2 knockout (C2), CMP2 double knockout, CARP3 knockout (C3) and CMP3 double knockout animals. Mean values and standard errors with p-values and sample sizes (n; displayed at the base of each bar) are shown. (E) Fractional shortening (FS) as calculated from

Figure 4C, representative for cardiac functions of 2- and 6-months old mice. Displayed are mean values and standard error with p-values and sample sizes (n; indicated in the base of each bar). (F) Quantitative PCR to test relative normalized (against GAPDH) mRNA levels of CARP1 in cardiac samples of WT, MLP knockout, CARP2 knockout (C2), CMP2 double knockout, CARP3 knockout (C3) and CMP3 double knockout mice. (G) Analysis of fluorescence intensity ratios of CARP1, CARP2 and α-actinin staining between the intercalated disks (ID) and the sarcomeres (S) of WT and MLP, or MLP and CMP1 sections from hearts of 6-9 months old mice (images analyzed as in Figure S1D). Displayed are mean values of ID/S ratios from 2-3 hearts per group, standard errors, sample size (n in base of bar graph) and p-values. (H) Quantification of immunofluorescence intensity (F-F0) of PKCα over IDs (as in Figure 2C) from WT, CARP1 knockout, MLP knockout and CMP1 double knockout heart sections. Sample sizes for analyzed ID, and p-values are indicated in the figure (please see the Materials and Methods section for a detailed description of the methodology). (I) Representative immunofluorescence images of WT, CARP1 knockout, MLP knockout and CMP1 double knockout used for the quantification in S2H. Cardiac cryosections were stained with antibodies against PKCα (green), plakoglobin (red) and α-actinin (blue). Scale bar = 10μm.

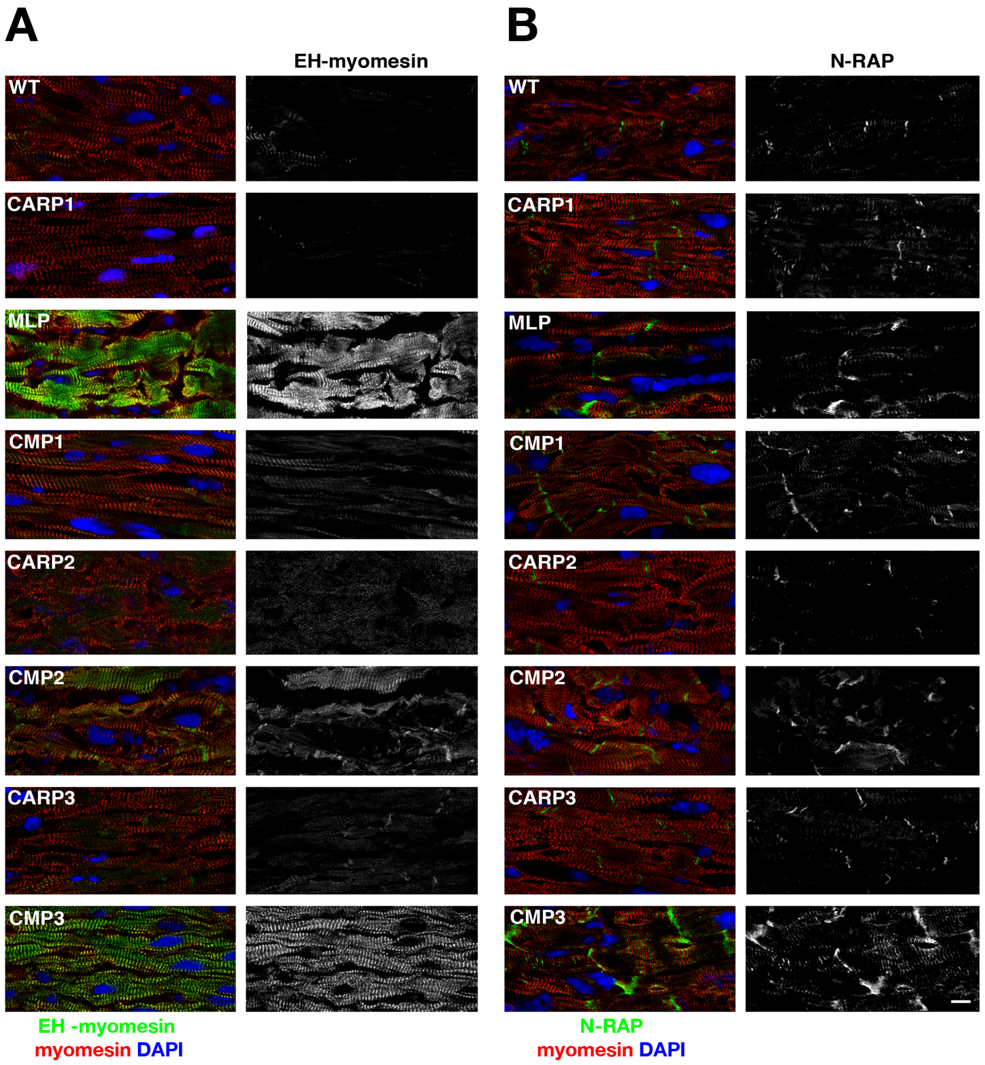

Supplementary Figure 3: Immunofluorescence staining of EH-myomesin (A; green in overlay) or nebulin-related anchoring protein (N-RAP; B; green in overlay) in frozen sections of adult hearts from WT, CARP1 knockout, MLP knockout, CMP1 double knockout, CARP2 knockout, CMP2 double knockout, CARP3 knockout and CMP3 double knockout animals. Myomesin (in red) and DAPI (in blue) were used as counterstains. Scale bar = 10 μm.

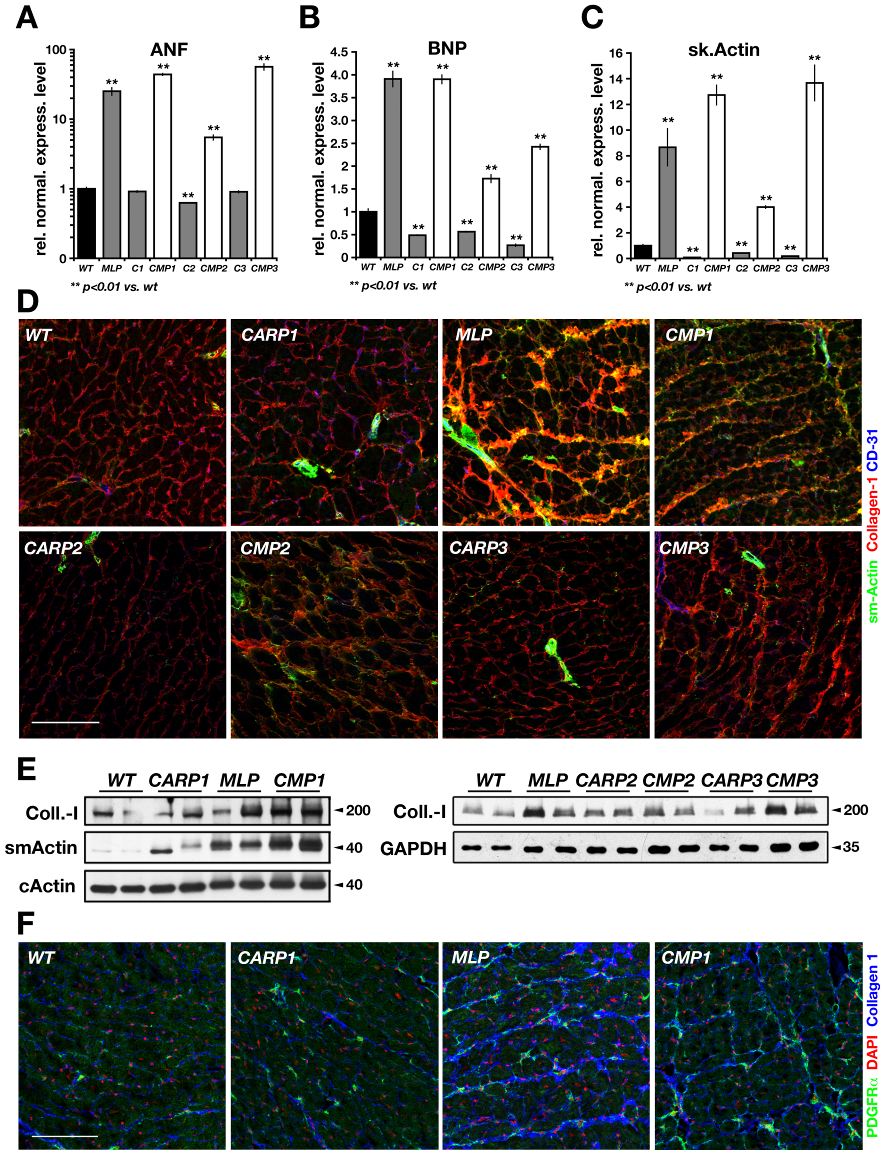

Supplementary Figure 4: (A-C) Normalized (vs. GAPDH) relative mRNA levels of fetal gene program markers ANF/ANP (A), BNP (B) and skeletal actin (skActin; C) of cardiac samples from WT, MLP knockout, CARP1 knockout (C1), CMP1 double knockout, CARP2 knockout (C2), CMP2 double knockout, CARP3 knockout (C3) and CMP3 double knockout mice as determined by quantitative-PCR. Displayed are mean values of deltadeltaCt expression-values from biological triplicates (n=3) and standard errors; p-values are indicated. Note logarithmic scale in (A). (D-F) Assessment of cardiac fibrosis and vascularization. (D) Immunofluorescence of representative frozen sections of left-ventricular free wall from WT, MLP knockout, CARP1 knockout, CMP1 double knockout, CARP2 knockout, CMP2 double knockout, CARP3 knockout, CMP3 double knockout animals using collagen-1 (red) and smooth muscle actin (sm-Actin; green). CD-31 counterstain (blue) was used to ascertain origin of increased sm-Actin staining. Note that there is no change in vascularization between MLP knockout and either CMP1 or CMP2 knockout hearts. Scale bar = 100μm. (E) Expression levels of soluble high molecular weight pro-collagen-1 and smooth muscle actin (sm-Actin) in whole heart extracts from wildtype, CARP1, MLP

and CMP1 double knockout mice (left panel), and of pro-collagen-1 in whole heart extracts from wildtype, MLP, CARP2, CMP2, CARP3, CMP3 mice (right panel). Cardiac actin (cActin) or GAPDH were used as loading controls, respectively. (F) Immunofluorescence using collagen-1 and platelet-derived growth factor receptor α (PDGFα) antibodies in representative frozen sections of free left ventricular wall from hearts of WT, MLP knockout, CARP1 and CMP1 animals. DAPI was used as counterstain. Scale bar = 100 μm.

Supplementary Table 1. Echocardiography analysis of wildtype, CARP1, MLP and CMP (CARP1 x MLP double knockout) mice at 2 months, 4 months, 6 months and 1 year of age. Shown are mean values and standard errors for fractional shortening (FS), the inter-ventricular septal thickness (IVSd), the left ventricular internal dimension (LVIDd, LVIDs), the left ventricular posterior wall thickness (LVPWd) during diastole and systole, respectively. Sample sizes (*n-values)* and *p*-values comparing the knockouts vs. wildtype are indicated, with $p < 0.05$ shown in italics, and $p < 0.01$ displayed in bold italics.

		FS (%)	IVSd (mm)	LVIDd (mm)	LVIDs (mm)	LVPWd (mm)
**2 months** *n=9 (WT, MLP) n=6 (CARP1, CMP)*	***WT***	46.0 ± 1.9	0.60 ± 0.01	3.51 ± 0.12	1.90 ± 0.11	0.59 ± 0.01
	CARP1	48.5 ± 1.0	*0.64 ± 0.01*	3.54 ± 0.11	1.82 ± 0.06	*0.61 ± 0.01*
	MLP	***25.7 ± 3.6***	0.59 ± 0.02	***4.23 ± 0.16***	***3.17 ± 0.24***	0.57 ± 0.02
	CMP	46.3 ± 2.4	0.61 ± 0.01	3.19 ± 0.08	1.71 ± 0.07	0.61 ± 0.01
**4 months** *n=6*	***WT***	43.4 ± 1.7	0.64 ± 0.01	3.41 ± 0.10	1.93 ± 0.07	0.61 ± 0.01
	CARP1	47.0 ± 1.6	0.64 ± 0.01	3.19 ± 0.14	*1.69 ± 0.06*	0.60 ± 0.01
	MLP	***16.8 ± 1.3***	***0.53 ± 0.01***	***4.72 ± 0.11***	***3.92 ± 0.10***	***0.53 ± 0.01***
	CMP	44.2 ± 2.0	*0.60 ± 0.01*	3.32 ± 0.08	1.85 ± 0.09	0.63 ± 0.02
**6 months** *n=6*	***WT***	43.3 ± 2.0	0.64 ± 0.02	3.47 ± 0.13	1.97 ± 0.09	0.63 ± 0.01
	CARP1	48.4 ± 2.0	0.63 ± 0.01	3.20 ± 0.14	*1.64 ± 0.07*	0.62 ± 0.01
	MLP	***16.4 ± 2.2***	***0.54 ± 0.01***	***4.93 ± 0.12***	***4.13 ± 0.19***	***0.56 ± 0.01***
	CMP	42.6 ± 2.5	0.64 ± 0.01	3.38 ± 0.20	1.96 ± 0.18	0.62 ± 0.01
**12 months** *n=3*	***WT***	49.5 ± 5.7	0.79 ± 0.08	3.68 ± 0.41	1.87 ± 0.43	0.74 ± 0.04
	CARP1	49.2 ± 2.8	0.70 ± 0.06	3.60 ± 0.54	*1.84 ± 0.37*	0.68 ± 0.09
	MLP	***17.5 ± 2.5***	***0.59 ± 0.03***	***5.11 ± 0.15***	***4.22 ± 0.09***	***0.59 ± 0.05***
	CMP	42.3 ± 4.8	0.66 ± 0.05	3.65 ± 0.08	2.11 ± 0.20	0.66 ± 0.03

Supplementary Table 2. Echocardiography analysis of wildtype, CARP2 and CMP2 (CARP2 x MLP double knockout) mice at 2 months and 6 months of age. Shown are mean values and standard errors for fractional shortening (FS), the inter-ventricular septal thickness (IVSd), the left ventricular internal dimension (LVIDd, LVIDs), the left ventricular posterior wall thickness (LVPWd) during diastole and systole, respectively. Sample sizes (*n*) and *p* values comparing the knockouts vs. wildtype (supplemental table 1) are indicated, with $p < 0.05$ shown in italics, and $p < 0.01$ displayed in bold italics.

		**FS (%)**	**IVSd (mm)**	**LVIDd (mm)**	**LVIDs (mm)**	**LVPWd (mm)**
**2 months**	***CARP2***	48.2 ± 0.6	*0.64 ± 0.01*	3.31 ± 0.19	1.72 ± 0.17	0.63 ± 0.01
n=3 (CARP2) n=5 (CMP2)	***CMP2***	39.2 ± 4.1	0.60 ± 0.03	3.92 ± 0.18	2.41 ± 0.26	0.61 ± 0.03
**6 months**	***CARP2***	47.5 ± 2.9	0.66 ± 0.01	***4.13 ± 0.06***	2.18 ± 0.14	0.64 ± 0.03
n=4 (CARP2) n=6 (CMP2)	***CMP2***	*34.1 ± 3.3*	*0.69 ± 0.02*	***4.20 ± 0.19***	***2.78 ± 0.23***	***0.70 ± 0.01***

Supplementary Table 3. Echocardiography analysis of wildtype, CARP3 and CMP3 (CARP3 x MLP double knockout) mice at 2 months and 6 months of age. Shown are mean values and standard errors for fractional shortening (FS), the inter-ventricular septal thickness (IVSd), the left ventricular internal dimension (LVIDd, LVIDs), the left ventricular posterior wall thickness (LVPWd) during diastole and systole, respectively. Sample sizes (*n)* and *p* values comparing the knockouts vs. wildtype (supplemental table 1) are indicated, with $p < 0.05$ shown in italics, and $p < 0.01$ displayed in bold italics.

		FS (%)	IVSd (mm)	LVIDd (mm)	LVIDs (mm)	LVPWd (mm)
**2 months** *n=6*	***CARP3***	44.5 ± 1.5	*0.64 ± 0.02*	3.60 ± 0.14	2.00 ± 0.11	0.61 ± 0.01
	CMP3	***26.5 ± 3.9***	0.61 ± 0.02	*4.11 ± 0.27*	***3.07 ± 0.35***	0.60 ± 0.02
**6 months**	***CARP3***	47.4 ± 1.3	0.66 ± 0.01	3.74 ± 0.23	1.97 ± 0.15	0.65 ± 0.02
n=3 (CARP3) n=6 (CMP3)	***CMP3***	***23.2 ± 5.0***	0.62 ± 0.05	***5.07 ± 0.16***	***3.93 ± 0.34***	0.62 ± 0.04

Supplementary Table 4. Oligonucleotides used for genotyping.

Mouse strain	Forward	Reverse
MLP Wildtype	CAGGCTGTCCCCTAGACCTC	GAACCACCAACAGACAGTAGTAGG
MLP Knockout	CCTTCTATCGCCTTCTTGACGAG	CTCATACTCGGAACTTGGG
CARP1 Wildtype	AATGTGGGGTTCGCGTTAGTGAC	GTCCTTCCTCTCGATCCAGCGACA
CARP1 Knockout	AACCTCGGCACATCCACAGGTTC	CTCATACTCGGAACTTGGG
CARP2 Wildtype	AACTTCGAAGATCCGCTCCTGG	CCTCAGTTCAATGAGGTTCTGGATC
CARP2 Knockout	AACTTCGAAGATCCGCTCCTGG	CTCATACTCGGAACTTGGG
CARP3 Wildtype	CCCTACCCTGTGGCTGATGTCGG	GGAGAGGGCCTCGGCAGCTAAGGG
CARP3 Knockout	CCCTACCCTGTGGCTGATGTCGG	CTCATACTCGGAACTTGGG
Gαq overexpressors	CAGGACTTCACATAGAAGCC	CGTGAAGATGTTCTGATACACC

Supplementary Table 5. Oligonucleotides for Q-PCR analysis.

**Name**	**sequence**
ANF.fwd	GATAGATGAAGGCAGGAAGCCGC
ANF.rev	AGGATTGGAGCCCAGAGTGGACTAGG
BNP.fwd	CCTCCGGGTCCAGCAGAG
BNP.rev	GGTCTTCCTACAACAACTTC
skeletal actin.fwd	GGCGGTGCTGTCCCTCTATG
skeletal actin.rev	TGTCGCGCACAATCTCACG
α-MyHC.fwd	CTGCTGGAGAGGTTATTCCTCG
α-MyHC.rev	GGAAGAGTGAGCGGCGCATCAAGG
β-MyHC.fwd	TGCAAAGGCTCCAGGTCTGAGGGC
β-MyHC.rev	GCCAACACCAACCTGTCCAAGTTC
S18.fwd	GGAAGGGCACCACCAGGAGT
S18.rev	TGCAGCCCCGGACATCTAAG
GAPDH.fwd	CTCAAGATTGTCAGCAATGCATCC
GAPDH.rev	CCAGTGGATGCAGGGATGATGTTC
CARP1.fwd	TCCGGCCAACAGCGTGAAG
CARP1.rev	TTCTGGCTCCTTCACAAC
